# Assessment of Industrially Produced Trans Fatty Acids in Traditional Dishes, Arabic Sweets, and Market Food Products and Its Risks on Non-communicable Diseases in Lebanon

**DOI:** 10.3389/fnut.2021.727548

**Published:** 2021-10-21

**Authors:** Maha Hoteit, Edwina Zoghbi, Alissar Rady, Iman Shankiti, Carla Ibrahim, Ayoub Al-Jawaldeh

**Affiliations:** ^1^PHENOL Research Group (Public Health Nutrition Program-Lebanon), Faculty of Public Health, Lebanese University, Beirut, Lebanon; ^2^Country Office for Lebanon, World Health Organization, Beirut, Lebanon; ^3^Faculty of Arts and Sciences, Department of Nutrition and Food Sciences, Holy Spirit University of Kaslik, Jounieh, Lebanon; ^4^World Health Organization Regional Office for the Eastern Mediterranean, Cairo, Egypt

**Keywords:** traditional dishes, Arabic sweets, market foods, Lebanon, industrially produced trans fatty acids

## Abstract

Industrially produced trans fatty acids (IP-TFAs) are a major dietary contributor to non-communicable diseases worldwide. To address the industrially produced trans fatty acids food sources in Lebanon, a mapping exercise was enrolled between January 2019 and April 2021 to establish a national database. The 2019 survey was a pooled data from five separate sources and had relatively 30 types of traditional dishes. In contrast, the subsequent surveys in 2020 had a sample of 35 types of Arabic sweets and 80 types of market food products. The 2021 survey covered all types of butter and margarine available in the Lebanese markets. Our findings show that about 93% of the products tested in Lebanon, between 2019 and 2021, met the World Health Organization recommendations, while about 7% exceeded the limit. The mean level of the IP-TFAs elaidic and linolelaidic acids in most traditional dishes (0.9%), Arabic sweets (0.6%), butter, and margarine (1.6%), and market foods (0.52%) were relatively low compared with other countries. Although trans fatty acids have a small impact on heart disease mortality in Lebanon, they are unquestionably significant. The persistence of food products with high quantities of trans fatty acids poses a health risk to Lebanese citizens. Fortunately, proper laws in Lebanon can easily remedy this situation.

## Introduction

In the past 50 years, the world has seen a dramatic shift in the causes of death and disabilities from infectious diseases and nutritional deficiencies to non-communicable diseases (NCDs) led by cardiovascular diseases (CVDs) (1), which causes an estimated 17.9 million deaths every year (2). As a result, the urgent need for policy measures to protect cardiovascular health is more apparent than ever and presents a historic imperative to prioritize and invest in public health by adopting health-promoting policy measures, including industrially produced trans fatty acids (IP-TFAs) elimination. Intake of IP-TFAs is associated with an increased risk of heart attacks and death from coronary heart disease (CHD) (3). A 2% absolute increase in energy intake (EI) from trans-fat has been associated with a 23% increase in cardiovascular risk (4). Although limited data are available on IP-TFAs intake globally, a recent report estimated that the 2017 global market volume of partially hydrogenated oils (PHOs), the main source of IP-TFAs in food, was ~13.6 million tones (5). PHOs constitute 25–45% of total fat (6). The most common non-conjugated IP-TFAs in the daily diet of humans are 18-carbon fatty acids with one double bond in the 9-carbon transposition or two double bonds in the 9 and 12 carbon, called elaidic acid (EA; 9t18:1) and linolelaidic acid (LEA; 9t12t18:2), respectively (7). EA, which is the *trans* form of oleic acid (OA, C18:1 *cis*), is the principal TFA found in partially hydrogenated vegetable oil and margarine. Following ingestion, EA is typically integrated into the plasma membrane of cells. EA has also been shown to induce oxidative stress; for example, an EA-rich diet caused oxidative stress in mice due to EA-induced reduction in plasma vitamin E levels (7). EA intake resulted in significant hyperlipidemia, inflammation, and fatty liver alterations (8). In addition, a recent study by Buyun Liu et al. found that plasma EA concentration is associated with a higher risk of depressive symptoms (9). Another study demonstrated that EA could change physiochemical surface properties of lactobacilli, an antimicrobial, cholesterol-lowering, immunoregulatory, and gut-beneficial bacteria (10). LEA is an omega-6 TFA (9E,12E-9t12t18:2), principally discovered in foods with fried or high-heat cooking or partially hydrogenated vegetable oils (11). It was suspected to enhance the adipogenic differentiation favoring obesity (11). Moreover, it was shown that LEA appeared to be potentially more detrimental than EA and that LEA contributed to higher risks of sudden cardiac death compared to other isomers (12). Furthermore, in a survey conducted by Qiu Bin et al., it is discovered that EA and LEA triggered endothelial cell apoptosis *via* the caspase pathway and the mitochondria pathway and that LEA induced higher human umbilical vein smooth muscle cell proliferation than EA (13). Around 58 countries have introduced laws to date that will protect more than 3 billion people from TFAs by the end of 2021 (14). However, more than 100 countries have yet to act to eliminate TFAs from their national food supply and make the world TFAs free by 2023 (14). The food industry is also becoming more receptive to replacing IP-TFAs in their products with healthier oils and fats. In 2018, TFAs elimination was identified as one of the priority targets in the WHO 13th General Programme of Work, which guides the 5-year work of WHO in 2019–2023 (14). Also in 2018, the REPLACE action package was launched to help countries in removing IP-TFAs from their food supplies (15). In addition, WHO released additional resources in 2019 to support country actions, including six implementation modules and a live policy tracking map, the TFAs Country Score Card 1, to monitor global progress toward the 2023 target (14). In 2020, WHO established a TFA indicator that records whether countries have adopted WHO best-practice policies for eliminating IP-TFAs (16). Their removal from the global food supply could prevent up to 17 million deaths by 2040 and would be the first time an NCD risk factor has been eliminated (17). The Eastern Mediterranean Region (EMR) has witnessed rapid modernization in the past 30 years that has led to a dramatic transformation affecting people's lifestyles and diet. The average intake of saturated fatty acids (SFAs) and TFAs in EMR exceeded the WHO upper limits and was estimated to be 10.3 percent and 1.9 percent of total EI, respectively (18). The highest SFAs intake was reported in Djibouti, Kuwait, Saudi Arabia, Lebanon, and Yemen, while the highest intake of TFAs was reported in Egypt and Pakistan. The main sources of TFAs in the region are margarine, biscuits, French fries, cereal-based foods, fast food, snacks, milk, bakery products, pie, and cake. Lower TFA content was reported in traditional foods (18). With a population estimate of around 6,803,105 million and a severe decline of the gross domestic product from the US $7,661 per capita in 2019 to the US $2,744 in 2020 (19), Lebanon is characterized by a high urbanization rate (88%) (20) coupled with westernization and modernization in diet and lifestyle and higher uptake of NCD risk factors. Similar to the region, the Lebanese population suffers from a high burden of NCDs, accounting for 91% of total annual deaths with CVDs responsible for 47% of total deaths (21). Because IP-TFAs increase the risk for heart disease and are estimated to cause more than 500,000 deaths per year (14) and based on the WHO recommendation that TFAs intake should not exceed 1% of total daily EI (equivalent to <2.2 g/day in a 2,000-calorie diet), providing baseline information on dietary sources of IP-TFAs in Lebanon is a crucial stepstone to reduce the risk of death and hospitalization by CVDs and is one of the strategic interventions under the area of prevention and reduction of risk factors in the Regional Framework for Action on NCDs (22). The main objectives of this article are to:

- Assess IP-TFA levels in frequently consumed traditional dishes, Arabic sweets, processed foods, butter, and margarine in Lebanon.
- Establish a stepping stone for required policies and regulations to mandate limits of TFA levels in oils and foods imported or produced locally.

## Materials and Methods

A series of surveys were conducted over the past 2 years. The 2019 survey was not centrally coordinated but instead pooled data from five separate sources having the broadest geographical coverage in terms of location and had relatively a sample size of 30 types of traditional dishes. The samples were identified according to their frequency of consumption (23, 24) and selected for TFA analysis along with two non-conjugated fatty acids (EA and LEA) (25). In contrast, the subsequent surveys 2020 were centrally coordinated having the broadest coverage in terms of products selected and had a sample of 35 types of Arabic sweets and 46 types of market food products. The full methodology of food list identifications and food sampling is described elsewhere (25–27). On the other hand, the 2021 survey was nationally coordinated, with a coverage of 34 types of butter, ghee, and margarine purchased from the Lebanese markets. Lot numbers were checked to ensure that each unit belonged to a different lot. The samples were stored, labeled, and analyzed before expiry dates. Samples were selected to include all types of butter, ghee, and margarine in Lebanon. The analyses were carried out in duplicate for each sample. To further interpret current levels of TFAs and partially hydrogenated fatty acids in Lebanese foods, product categories were compared to similar products found in other countries.

### Laboratory Analysis Protocol

After the receipt of food samples by the laboratory, 500 g of each sample was mashed, then analyzed. The remaining samples were kept frozen at −18°C in tight containers for further analysis. The fatty acid profile was measured using gas chromatography. Each analysis method was selected considering guidance from the technical committee at the Industrial Research Institute laboratories in Beirut and following standardized protocols. The Association of Official Analytical Chemists (AOAC) methods were used for the analysis of nutrients in food matrices (28). The full details about the measurement techniques are listed in the study described by Hoteit et al. (27), Association of Official Analytical Chemists (28), and Hoteit et al. (29).

The sum of trans fatty acids was calculated accordingly (28). TFA isomers were later on detected through SP-2560 100-m capillary column (180°C isothermal, H2 at 1.0 mL/min) (30).

## Results

### Trans Fatty Acid Acids in Frequently Consumed Traditional Dishes

#### Appetizers, Pastries, and Composite Dishes by Governorates

The mean levels of IP-TFAs in the traditional dishes among all governorates together (average:0.9%) ranged from <0.1 to 1.9 g/100 g of total fat except the dishes *Riz a dajaj* and *Shawarma Lahma*, which contained an amount of EA and LEA that both exceeded 2% of fat (Table 1). The comparison between the mean values of the IP-TFA (EA and LEA) in the traditional dishes tested shows that EA was significantly higher than LEA in all traditional dishes (*p* = 0.00). Per each governorate, the mean level of IP-TFAs in all dishes was 0.4% in Mount Lebanon (average: 0.4%; range: <0.1–2.1%), 1.1% in Beqaa (average: 1.1%; range: <0.1–2.3%), 0.8% in Beirut (average: 1.1%; range: <0.1–11.2%), 1.5% in Tripoli (average: 1.5%; range: <0.1–10.6%), and 0.8% in Saida (average: 0.8%; range: <0.1–2%) (data not shown). Moreover, per each governorate, the mean level of EA was 0.6% in Mount Lebanon, 0.9% in Beqaa, 0.9% in Beirut, 1.2% in Tripoli, and 0.6% in Saida (data not shown).

**Table 1:** Mean levels of total fat in 100 g of edible portions, total IP-TFAs, and IP-TFAs (EA and LEA) in 100 g of fat of frequently consumed traditional dishes among all Lebanese governorates.

			**IP-TFAs in 100 g of total fat**	
**Dish**	**Total fat (g) in 100 g**	Total IP-TFA^*^ per 100 g of total Fat	**Trans-C18:1n9t (Elaidic acid)**	**Trans-C18:2n6t (Linolelaidic acid)**
Baba ghanouj	9.44	0.74	0.66	0.08
Batata mahchi	1.24	1.98	1.92	0.06
Borgul bil banadoura	5.02	0.48	0.34	0.14
Chichbarak	4.62	0.98	0.86	0.12
Falafel	11.70	0.36	0.32	0.04
Fatayer sabanikh	11.16	0.18	0.12	0.04
Fattat Hommos	7.04	0.66	0.58	0.08
Fattoush	2.94	0.8	0.5	0.3
Foul moudamas	3.48	0.46	0.38	0.08
Hindbe bil zet	10.70	0.18	0.18	0
Hommos bi tahini	6.44	0.38	0.24	0.14
Kafta wa batata	6.32	1.28	1.18	0.1
Kebba bil sayniya	6.40	0.86	0.74	0.12
Koussa mahchi	2.42	1.26	1.1	0.16
Lahm bil ajin	8.96	0.34	0.22	0.12
Loubia bil zet	5.68	0.52	0.46	0.06
Malfouf mahchi	2.12	1.1	1.02	0.1
Moujadara	5.80	0.36	0.36	0
Moghrabia	3.94	0.86	0.76	0.1
Mousaka batinjan	6.58	0.5	0.34	0.16
Riz a dajaj	5.42	2.82	2.66	0.16
Riz bi lahma	6.52	0.82	0.78	0.04
Sayadia	6.48	0.22	0.18	0.04
Shawarma dajaj	6.94	0.24	0.16	0.08
Shawarma lahma	8.28	2.24	2.08	0.16
Tabboula	4.24	0.38	0.26	0.12
Warak enab	3.98	1.24	1.06	0.18
Yakhnat Bamia	5.42	1.24	1.02	0.22
Yakhnat Fassoulia	3.90	0.76	0.64	0.12
Yakhnat Mouloukhia	4.28	1	0.8	0.2

* *This represents the sum of EA and LEA only*.

### Trans Fatty Acid Acids in Frequently Consumed Arabic Sweets

The total average of IP-TFAs in all samples of Arabic sweets was 0.6%, predominantly from EA type (Table 2). Among 35 samples of Arabic sweets, none exceeded 2% as total IP-TFA in 100 g of total fat. The comparison between the mean values of the IP-TFA (EA and LEA) in the Arabic sweets tested shows that EA was significantly higher than LEA in Arabic sweets (*p* = 0.00).

**Table 2:** Mean levels of total fat in 100 g of edible portions, total IP-TFAs, and IP-TFAs (EA and LEA) in 100 g of total fat of frequently consumed Arabic sweets.

			**IP-TFAs in 100 g of total fat**	
**Name**	**Total fat (g) in 100 g**	**Total IP-TFAs per 100 g of total fat**	**Trans-C18:1n9t (Elaidic acid)**	**Trans-C18:2n6t (Linolelaidic acid)**
Baklava mixed	23.45	0.25	0.2	0.05
Baklava mixed light	20.5	0.3	0.3	0
Halawat El Jiben	8.95	1.2	1.05	0.15
katayef Kashta	6.65	0.9	0.65	0.25
Kounafa bil jiben	12.25	0.4	0.25	0.15
Maakaroun	12	0.1	0.1	0
Maamoul Tamer	17.4	0.4	0.25	0.15
Maamoul mad Kashta	10.65	0.4	0.25	0.15
Maamoul mad joz	19.2	0.45	0.4	0.05
Maamoul joz	21.5	0.85	0.75	0.1
Mafrouka Kashta	13.25	0.4	0.2	0.2
Mafroukeh fostok	10.6	0.6	0.5	0.1
Moushabak	20.1	0.4	0.4	0
Nammoura	5.9	1.5	1.3	0.2
Osmaliya	16.25	0.5	0.4	0.1
Saniora	23.8	1.15	0.85	0.3
Sfouf	12.45	1.45	1.2	0.25
Barazik	16.5	0.5	0.5	0
Boundoukia	19.5	0.3	0.3	0
Daoukia	14.8	0.4	0.3	0.1
Foustoukia	20.4	0.4	0.4	0
Ghourayba	25.8	0.6	0.4	0.2
Ish el bulbul	25.1	0.2	0.2	0
kallaj kashta	9.6	<0.1	-	-
Karabij joz maa crema	18.8	0.4	0.2	0.2
kounafa kashta maa kaak	10	0.4	0.1	0.3
Maakroun wa Moushabak	13.7	<0.1	-	-
Maamoul fostok	19.1	0.7	0.5	0.2
Madlouka	11.5	0.6	0.5	0.1
Moufattaka	20.7	<0.1	-	-
Mouhallabiya	4	0.5	0.1	0.4
Riz bil Halib	4.4	<0.1	-	-
Shaaybiyat	16.1	<0.1	-	-
Ward el sham	14.2	0.5	0.5	0
Znoud El sitt	12.3	<0.1	-	-

*This represents the sum of EA and LEA only.

### Trans Fatty Acid in Market Foods

#### Cereals and Bread Group

In the group of cereals and bread, the mean level of IP-TFAs was <2% of total fat except for *pain au lait*, which is usually prepared from wheat, milk, and butter or ghee to be consumed frequently by children as a sandwich (Table 3).

**Table 3:** Total fat in 100 g of edible portions, total IP-TFAs, and IP-TFAS (EA and LEA) in 100 g of total fat of market food products collected from Lebanese markets.

			**IP-TFAs in 100 g of total fat**	
**Product**	**Total fat (g) in 100 g**	**Total IP-TFAs per 100 g of Total Fat**	**Trans-C18:1n9t (Elaidic acid)**	**Trans-C18:2n6t (Linolelaidic acid)**
Arabic bread-white	2.3	<0.1	–	–
Arabic bread-whole wheat	4	<0.1	–	–
Baguette	0.5	<0.1	–	–
Biscuits chocolate quinoa	13.4	0.1	0.1	–
Biscuits digestive	17.1	0.3	0.1	0.2
Biscuits digestive light	13.8	0.3	0.1	0.2
Biscuits with cream	15.5	<0.1	–	–
Breakfast cereals	2.1	<0.1	–	–
Breakfast cereals-chocolate	2.4	0.3	0.1	0.2
Butter (*n =* 17 samples)	80	0.8	0.6	0.2
Cake with cream	16.1	<0.1	–	–
Chocolate dark	33.6	<0.1	–	–
Chocolate Milk-1	36.6	0.1	0.1	–
Chocolate Milk-2	35	<0.1	–	–
Coffee without cardamon	16.8	0.2	0.1	0.1
Coffee with cardamon	17.7	0.3	0.1	0.2
Corn oil	100	<0.1	–	–
Croissant Zaatar-1 (cheap)	16.1	0.7	0.4	0.3
Croissant zaatar-2 (expensive)	22.5	0.1	0.1	
De-hulled pumpkin seeds	50.6	0.6	0.3	0.3
De-hulled sunflower seeds	52.5	0.7	0.3	0.4
Doughnuts	19.6	0.5	0.5	–
English cake-chocolate	18.6	2.6	2.6	–
Margarines (*n =* 18)	100	2.4	2.2	0.2
Halawa	25.5	0.4	0.4	–
Halawa light	29.9	1.3	1.1	0.2
Hot chocolate powder	5.4	0.3	0.3	–
Instant coffee	10.8	0.2	0.2	–
Kaak asrouni	1.5	Tr	–	–
Kaak debes and cacao	11.9	0.3	0.2	0.1
Kaak korshalli	6.9	0.5	0.5	–
Mixed kernels	53.6	<0.1		–
Mixed nuts	25.7	0.3	0.2	0.1
Olive oil	100	<0.1	–	–
Pain au Lait	3.8	2.7	2.7	–
Petit Fours-1 (cheap)	25.6	0.2	0.2	–
Petit Fours-2 (expensive)	29.6	0.2	–	0.2
Potato Chips-1	29.9	0.1	0.1	–
Potato Chips-2	15.4	0.3	0.2	0.1
Potato Chips Light-1	26.9	0.1	0.1	–
Potato chips light-2	22.9	0.3	0.3	–
Sunflower oil	100	<0.1	–	–
Tahina	59.4	0.1	-	0.1
Tuna packed in oil	6.8	0.3	0.1	0.2
Tuna packed in water	0.5	0.6	0.6	–
Wafer-Chocolate-1	21.7	<0.1	–	–
Wafer-Chocolate-2 (manufactured in Lebanon)	24.2	6.5	6.2	0.3

#### Butter and Margarine

**Figure 1:**
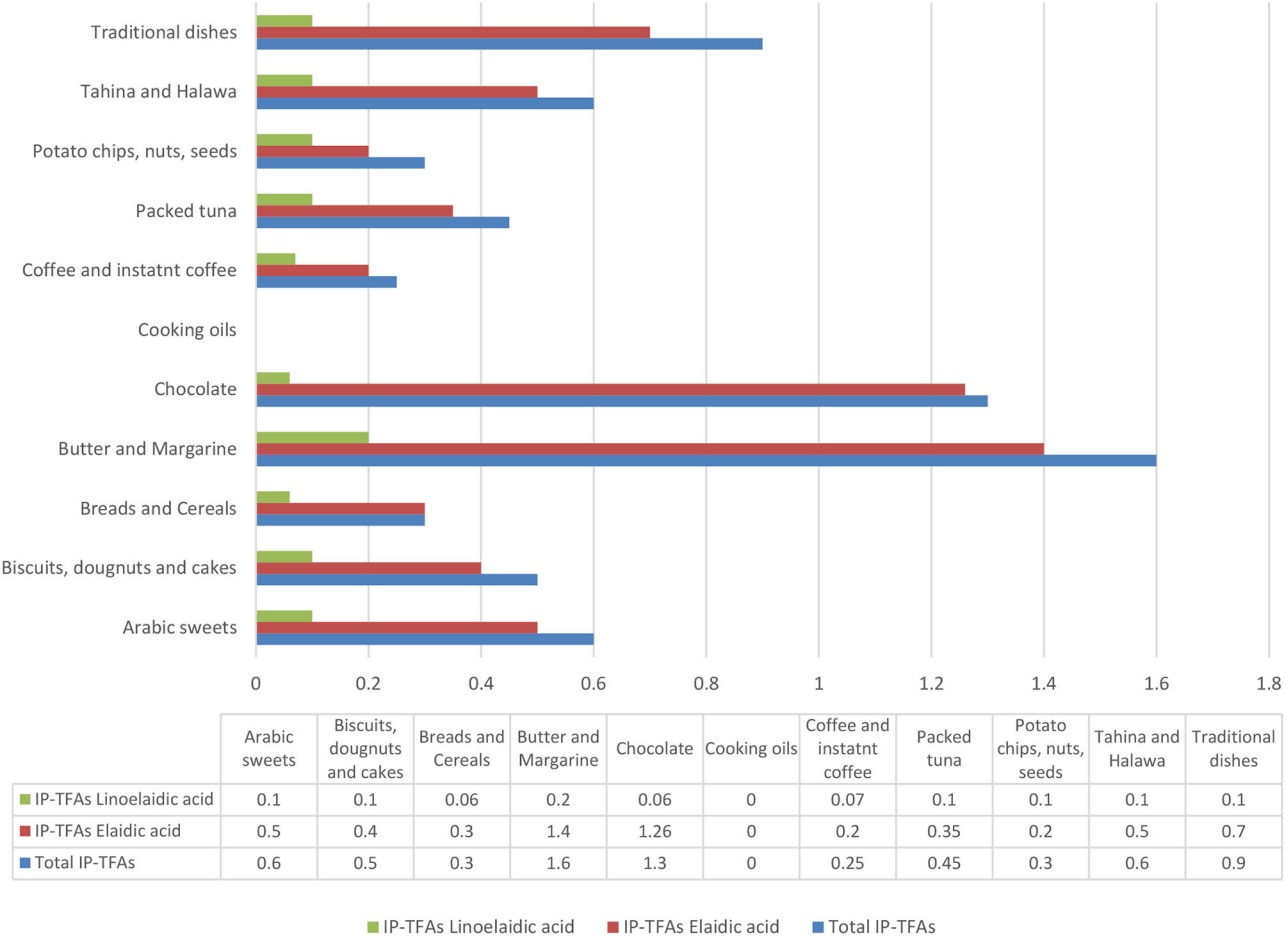
Total IP-TFAs and IP-TFAs averages in all Lebanese food groups tested in this mapping exercise.

Particular attention was given to the butter and margarine group as these are used as ingredients, and therefore among the main sources of TFAs in processed foods. The average of total IP-TFAs in 18 margarine used frequently in Lebanon was 2.4% (Table 3) with a range between <0.1 and 11.8% (data not shown). The dominant IP-TFA was EA in almost all these products (Table 3). Within the group of butter, none of the samples exceeded 2% of total fat. The average of total IP-TFAs in the butter and margarine group was 1.6% of total fat, in which EA predominates in these products. In general, the level of total IP-TFAs in cooking oils, Halawa and Tahina, was negligible (Table 3, Figure 1). In addition, Figure 1 shows that the average of total IP-TFAs was equal to 1.6% in the group of butter and margarine.

#### Snacks and Processed Foods

As for the group of biscuits, doughnuts, and cakes group, negligible amounts of IP-TFAs were found in these products (average: 0.5%) (Figure 1). On the other hand, the unlabeled English cake (chocolate flavor) had an apparently high amount of total IP-TFAs (2.6% in the total fat), in which EA was dominantly available (Table 3). Despite being unable to discuss the fat type used in unlabeled samples, based on these data, partially hydrogenated fats were certainly present in high amounts.

The data on chocolate products presented an amount of 1.3% as total IP-TFAs (Figure 1), except for the case of wafer-coated chocolate originally manufactured in Lebanon, which contains a level of 6.5% (Table 3).

According to Table 3, Figure 1, it appears that all samples of potato chips, nuts, seeds, coffee, instant coffee, and packed tuna contained low amounts of total IP-TFAs that are below 2% of total fat.

When comparing the mean values of the IP-TFAs (EA and LEA) in the market foods, EA and LEA did not show any significant difference (*p* = 0.16).

### Comparison Between Market Foods Nutrient Content and Nutrition Facts Label

All the products were found to have discrepancies in reporting the actual nutrient content when compared to their respective nutrition facts label (Table 4). For instance, some products such as sunflower and olive oil had matching values for fat content. As for the SFAs content, there was a discrepancy between the nutrition label and nutrient content of the chocolate wafers-brand 2 (19.1/100 g and 9/100 g), chocolate with milk (36.1/100 g and 46/100 g), chocolate-dark (33.1/100 g and 23.3/100 g), and potato chips (20.2/100 g and 10/100 g). With regard to TFAs, it was either mentioned as 0 g or not reported on the nutrition label, while analysis showed traces of TFAs in the foods (Table 4). The comparison between the mean values of fat, SFA, and TFA reported on the label and that analyzed in the laboratory showed no major differences (*p* = 0.77, 0.173, and 0.264, respectively).

**Table 4:** Comparison between actual nutrient analysis values of some market-processed food products and reported nutrient values on nutrition fact label.

**Market Food Product**	**Fat**		**Saturated fatty acids**		**Trans fatty acids**	
	Chem-R^*^	**N. label^∧^**	**Chem-R**	**N.label**	**Chem-R**	**N.label**
Biscuits chocolate quinoa	13.4	5	1.8	Tr	Tr	0
Biscuits digestive	17.1	26	15.6	15	Tr	0
Biscuits digestive light	13.8	13.5	12.2	13.5	Tr	0
Breakfast cereals	2.1	0.4	0.9	0.1	Tr	0
Breakfast cereals-chocolate	2.4	5.2	2.1	5.2	Tr	0
Butter	81.4	80	56.4	54	0.4	6.6
Butter light	61.5	20	43.4	53.3	0.4	0
Chocolate wafers	21.7	25	19.6	21	Tr	0
Chocolate wafers-brand 2	24.2	27.27	19.1	9	1.5	4.5
Chocolate with milk	36.6	73	36.1	46	0	–
Chocolate-dark	33.6	40	33.1	23.3	Tr	0
Chocolate-white	35	–	34.7	–	Tr	–
Corn oil	100	100	10.3	14	Tr	0
Cream filled biscuits	15.5	18.6	7.8	4.6	Tr	–
Hot chocolate powder	5.4	5.9	3.5	3.7	Tr	–
Instant coffee	10.8	9.09	10.6	9	Tr	–
Chips baked	22.9	22	15	6	Tr	0
Lays chips cheese	15.4	12	6.5	3	Tr	0
Olive oil	100	100	14.9	24	Tr	0
Potato chips	29.9	26.6	20.2	10	Tr	–
Potato chips light	26.9	20	18.3	6.6	Tr	0
Sunflower oil	100	100	7.6	10	Tr	0
Tahina	59.4	60	17.1	10	Tr	0
Tuna packed in oil	6.8	26.6	3.2	–	Tr	–
Tuna packed in water	0.5	3	0.2	–	Tr	–
Vegetable margarine	>99	56.4	46.7	9.5	Tr	–

* *Chem R, Chemical results*;

∧ *N. label: Nutrition label*.

## Discussion

### Industrially Produced Trans Fatty Acid Content in Frequently Consumed Foods in Lebanon Compared With Different Countries

The available data demonstrate that categories with the highest IP-TFA levels included *Riz a dajaj, Shawarma Lahma, Pain au lait, English cake, Chocolate wafers, and margarine*. About 93% of the products tested in Lebanon, between 2019 and 2021, met the WHO recommendations (less than 2% of trans fatty acid in total fat), while about 7% exceeded the limit. As per Tables 1–3, all in all, EA was dominant in almost all the analyzed samples, and its higher amount indicates that hydrogenated oils were a major contributor in the processing of food products or baking and cooking meals. In comparison with other countries all over the globe, a broad range of EA was observed in many food products (Table 4). For instance, the mean level of EA in Baklava (0.2%) was relatively low in our study in comparison with the content of EA in Iran (2.5%) (31). Furthermore, our findings showed that the mean levels of EA in cakes (2.6%) were much lower than the content of EA found in cakes in France (18.5–25.6%) (32), Iran (6.95–18%) (31–33), Poland (7.95%) (34), India (1.92–3.93%) (35), and higher than EA cake content tested in Lebanon in 2015 (1.7%) (36), Korea (1.36%) (37), Turkey (0.37%−1.43%) (38), New Zealand (0.9%) (39), and Malaysia (<0.001%) (40) (Table 4). In addition, the mean levels of EA in biscuits in Iran (9–12.86%) (33), Lebanon 2015 (3.7%) (36), Poland (2.81%) (34), Korea (2.4%) (37), New Zealand (0.9%) (39), and Germany (0.18%) (41) were higher than our results (0.1%), except for Malaysia (<0.001%) (40) and India (0.01%) (35) (Table 4). As for the breakfast cereals, the mean level of EA in our study (0.1%) was much lower than in France (28.9–32.4%) (32) and Korea (0.5–6.75%) (37), and higher than in the UK (0.03%) (42) and Malaysia (<0.001%) (40) (Table 4). Moreover, our finding showed that the mean level of EA in chocolate wafers was 6 times more than EA content in chocolate wafers in Malaysia (40). As for the butter, the New Zealand (39) and Costa Rican butter (43) contained five times more EA, and the Pakistani butter (44) contained three more times EA, compared with our results (Table 4). However, the butter in UK, Germany, and Iran contained 0.22% (42), 0.23% (41), and 0.3% (33) EA, respectively; this is lower than the content of EA tested in our study (0.6%) (Table 4). Also, Table 4 shows that the margarine in Slovenia contained the highest content of EA (34.63%) (45) compared to our findings (2.2%) and other countries. As for the EA content in chips, Iranian chips showed the highest level of EA (10%) compared to our results (0.1–0.3%) and other countries (33) (Table 5). On the other hand, the results of LEA in the food products tested in our study and those in other countries are available in Table 5.

**Table 5:** Industrially produced trans fatty acids (EA and LEA) in 100 g of total fat per food group among different countries.

	**IP-TFAs**	
**Countries/year**	**Trans-C18:1n9t (Elaidic acid) (%)**	**Trans-C18:2n6t (Linolelaidic acid) (%)**
France 1995–1996 Wolff et al. (32)	Cake: 24.43 Rolled cake: 25.6 Cereals: 28.9 Roasted bread: 33.1 Toasted bread: 25.8 Bread: 30.3 Brioche: 34.15 Crackers: 20.9 Corn flour: 26.8 Puff pastry: 23 Cookies 38.9	**–**
France 1999 Wolff et al. (32)	Melba toast: 40.6 Sandwich: 21.3 Muesli: 32.4 Crackers: 21.35 Cake: 18.5	**–**
New Zealand 1998 Saunders et al. (39)	Margarines and table spreads (low trans): 0.1 Margarines and table spreads: 12.3 Margarine/butter blends: 8.3 Butters: 5.2	Margarines and table spreads (low trans): 0.1 Margarines and table spreads: 1.3 Margarine/butter blends: 1.6 Butters: 1.7
Spain 2000 Alonso et al. (46)	Spanish margarines: 8.17	Spanish margarines: 0.49
Bulgaria 2002 Marekov et al. (47)	Imported margarines: 8.4 Bulgarian margarines: 1.12 Frying fats: 13.42	**–**
Turkey 2002 Tekin et al. (48)	Margarine tub: 3.85 Margarine stick: 16.88	Margarine tub: 0 Margarine stick: 2.09
Korea 2005 Lee et al. (37)	Breakfast cereal: 6.75 Cream-filled biscuit: 15.57 Cream-stuffed cake: 20.96 Canned coffee: 2.3	Breakfast cereal: 0.25 Cream-filled biscuit: 0.43 Cream-stuffed cake: 0.66 Canned coffee: 0.3
New Zealand 2006 Saunders et al. (39)	Biscuits and cakes: 0.9 Margarines/spreads: 4.9 Chocolate: 1.1 Snack bars: 0.4 Pies and pastry: 3.7 Partially cooked chips/wedges: 2.5	Biscuits and cakes: 0 Margarines/spreads: 0.1 Chocolate: 0 Snack bars: 0.1 Pies and pastry: 0.4 Partially cooked chips/wedges: 0.4
Pakistan 2006 Anwar et al. (44)	Margarines: 7.89 Butter: 3.82	Margarines: 0.45
Turkey 2006 Karabulut and Turan (49)	Margarines and shortenings: 10.55	–
Canada 2007 Ratnayake et al. (50)	Tub margarines: 3.4 Print margarines: 5.5	Tub margarines: 0.1 Print margarines: 0.3
Costa Rica 2007 Baylin et al. (43)	Corn oil: 0.35 Sunflower oil: 0.28 Olive oil: 0.26 Margarines: 10.15 Butter: 5.1 Mixed nuts: 0.2 Mayonnaise: 0.13 Canned tuna (oil): 0.54 Canned tuna (water): 1.07 Non-dairy coffee creamer: 30.84	Corn oil: 0.07 Sunflower oil: 0.09 Olive oil: 0 Margarines: 0.35 Butter: 0.23 Mixed nuts: 0 Mayonnaise: 0.02 Canned tuna (oil): 0.08 Canned tuna (water): 0 Non-dairy coffee creamer: 1.15
Korea 2008 Lee et al. (37)	Breakfast cereal: 0.5 Cream-filled biscuit: 2.4 Cream-stuffed cake: 1.36 Canned coffee: 2.3	Breakfast cereal: 0.3 Cream-filled biscuit: 0.25 Cream-stuffed cake: 0.26 Canned coffee: 0.7
Pakistan 2008 Kandhro et al. (51)	Margarines: 19.48	Margarines: 0.49
Brazil 2011 Suzuki et al. (52)	Regular dark Chocolate: 0.078 Regular chocolate: 0.075 Diet chocolate: 0.06	–
Germany 2011 Kuhnt et al. (41)	Margarines/spreads: 0.2 Shortenings/cooking fats: 0.51 French fries/chips: 0.27 Croquettes: 0.09 Puff pastries: 0.86 Doughnuts: 2.07 Chocolate products: 0.44 Biscuits: 0.18 Instant products: 0.36 Butter: 0.23	–
Mexico 2011 Hernández-Martínez et al. (53)	Spreadable margarines: 4.73 Stick margarines: 7.4	Spreadable margarines: 0.39 Stick margarines: 0.94
Turkey 2011 Cakmak et al. (38)	Potato crisps: 0.13 Corn crisps: 0.24 Cocoa cakes: 0.37 Mosaic cakes: 1.39 Chocolate cakes: 0.55 Cream cakes: 0.78 Hazelnut-cocoa cakes: 1.43 Fruity cakes: 1	Potato crisps: 0.15 Corn crisps: 0.16 Cocoa cakes: 0.11 Mosaic cakes: 0.05 Chocolate cakes: 0.08 Cream cakes: 0.24 Hazelnut-cocoa cakes: 0.14 Fruity cakes: 0.09
India 2012 Reshma et al. (35)	Biscuit: 0.01 Pastry: 0.85 Cake: 1.92 Bread: 0.18 Bun: 1.31 Puff: 3.02 Roll: 0.12	Biscuit: 0 Pastry: 0 Cake: 0.04 Bread: 0.007 Bun: 0.03 Puff: 0.01 Roll: 0
Iran 2012 Nazari et al. (33)	Cakes: 18 Cream biscuits: 12 Simple biscuits: 9 Simple chocolates: 5 Cream chocolates: 2 Potato chips: 10 Puffy: 13 Animal butter: 0.3 Margarine: 3.2	Cakes: 0 Cream biscuits: 2 Simple biscuits: 2 Simple chocolates: 0 Cream chocolates: 0 Potato chips: 4 Puffy: 7 Animal butter: 0.3 Margarine: 0.9
UK 2013 Roe et al. (42)	Breakfast cereal products: 0.03 Garlic and herb baguette, baked: 0.16 Fat spread (26–39% fat): 0.09 Fat spread (41–62% fat): 0.11 Fat spread (62–75% fat, not polyunsaturated): 0.07 Margarine, hard block: 0.05 Compound cooking fat: 0.06 Potato chips, takeaway: 0.97 Potato chips, fine cut, takeaway: 0.08 Potato chips, oven baked: <0.02 Potato snacks and corn snacks: 0.08 Confectionery, non-chocolate: 0.05 Confectionery, chocolate: 0.08 Chocolate spread: 0.03 Mayonnaise: <0.02 Baby rusks: 0.05 Butter, spreadable: 0.22	–
Malaysia 2013 Akmar et al. (40)	Cakes: <0.001 Doughnuts: <0.001 Croissants: <0.001–0.02 White bread: <0.001 Whole grain bread: <0.001 Buns: <0.001 Cream crackers: <0.001–0.33 Chocolate biscuits: <0.001 Potato chips: <0.001–0.87 Chocolate bars: <0.001 Chocolate wafers: <0.001–0.38 Olive oil: 0.79 Blended oil (canola, soybean and olive): 0.82 Soybean oil: 1.76 Palm oil: 1.79 Corn oil: <0.001 Mayonnaise: <0.001 Shortening: <0.001 Coco-coated cereal: 1.57	Cakes: <0.001 Doughnuts: <0.001 Croissants: <0.001 White bread: 3.12 Whole grain bread: <0.001 Buns: <0.001–1.21 Cream crackers: <0.001 Chocolate biscuits: <0.001–0.02 Potato chips: <0.001–1.02 Chocolate bars: <0.001–0.54 Chocolate wafers: <0.001 Olive oil: <0.001 Blended oil (canola, soybean and olive): 3.24 Soybean oil: 4.06
	Honey-coated cereal: <0.001 Corn cereal: <0.001 Cereal beverages: <0.001	Palm oil: <0.001 Corn oil: 2.13 Mayonnaise: <0.001–0.65 Shortening: <0.001–2.19 Coco-coated cereal: <0.001 Honey-coated cereal: 1.76 Corn cereal: 4.82 Cereal beverages: <0.001–6.60
Iran 2013 Hajimahmoodi et al. (54)	Liquid frying oils:0.08 Solid frying oils: 1.26	Liquid frying oils: 0.01 Solid frying oils: 0.03
Saudi Arabia 2013 Bakeet et al. (55)	Margarines and shortenings: 5.43	Margarines and shortenings:1.49
Iran 2014 Hajimahmoodi et al. (56)	Margarines:5.99	Margarines: 0.66
Iran 2015 Bahrami et al. (31)	Creamer:13.94 Biscuit: 12.86 Cake: 6.95 Shortcake: 3.38 Donuts: 3.29 Bread tan: 2.99 Baklava: 2.5 Chocolate: 1.24 Chips: 0.61 Snack: 0.52	–
Iran 2016 Abedi et al. (57)	Edible oils: 0.07 Margarines: 5.3	-
India 2017 Shaik et al. (58)	Cakes: 3.93	Cakes: 2.82
Lebanon 2015 Saadeh et al. (36)	Cakes: 1.7 Biscuits: 3.7 Croissant:2.7 Wafers: 5.6	Cakes: 0.1 Biscuits: 0.1 Croissant:0.1 Wafers: 0.1
Slovenia 2018 et al. (45)	Margarines and shortenings: 34.63	Margarines and shortenings: 21.38
Serbia 2018 Jasmina et al. (59)	Crackers: 0.9 Bake rolls and kubz: 0.3 Salted sticks: 6.6 Chips and flips: 5.34 Fried corn nuts: 1.7 Roasted soybean: 0.2 Expanded rice and rice cakes: 3.4	Crackers: 0.5 Bake rolls and kubz: 0.1 Salted sticks: 0.9 Chips and flips: 0.152 Fried corn nuts: 0.1 Roasted soybean: 0.05 Expanded rice and rice cakes: 2.7
Poland 2019 Zbikowska et al. (34)	Short crust: 0.95 Biscuits: 2.81 French pastry cookies: 1.65 Sponge cakes: 7.95	Short crust: 0.16 Biscuits: 0.21 French pastry cookies: 0.275 Sponge cakes: 0.44
Tunisia 2019 Selmi et al. (60)	Margarines: 4.47 Frying oil: 0.14	Margarines: 4.47 Frying oil: 0.24

### Trans Fatty Acid Content in Frequently Consumed Foods in Lebanon Compared to Eastern Mediterranean Countries

Compared to other countries, the average of TFAs in Arabic sweets (0.6/100 g of total fat), bakery products (0.6/100 g of total fat), and biscuits (0.17/100 g of total fat) in our study was relatively low compared to Jordan (4.08, 2.46, and 2.82/100 g of total fat, respectively) (61). Furthermore, the average of TFAs in biscuits in Iran in 2015 (12.86/100 g of total fat) (31), Lebanon in 2015 (9.7/100 g of total fat) (36), and Pakistan (9.3/100 g of total fat) (62) was higher than the value reported in our current study. As for butter and margarine, the highest content of TFAs was observed in Pakistan (9.3–34.9 /100 g of total fat) (62) followed by Morocco (9.1–21.7/100 g of total fat) (63) and the lowest level was observed in our current report. Similarly, cakes in Pakistan contained also the highest level of TFAs (12.02/100 g of total fat) (62) compared to Iran (2015) (6.9/100 g of total fat) (31), Lebanon in 2015 (5.8/100 g of total fat) (36), Jordan (3.4/100 g of total fat) (61), and our current findings (1.3/100 g of total fat). It was observed that cereals and bread tested in Pakistan (14.4/100 g of total fat) (62) contained the highest levels compared to our study (0.15/100 g of total fat) and Jordan cereal-based foods (2.5/100 g of total fat) (61). Furthermore, a decrease in TFAs was observed between the “croissant” tested in this report (0.4/100 g of total fat) and the samples tested in Lebanon 6 years ago (7.8/100 g of total fat) (36). Compared to Iran (2013 and 2016) (0.45–0.72/100 g of total fat) (54–57), to Jordan (0.61/100 g of total fat) (61), to Pakistan (0.96/100 g of total fat) (62), and Tunisia (0.73/100 g of total fat) (60), edible oils tested in our report were TFAs free. Chocolate samples and wafers in our study presented a broad range of TFAs. In the chocolate samples tested currently, the average of TFAs was 0.03%, which was lower than the values reported in Iran (1.24/100 g of total fat) (31) and Pakistan (4.5/100 g of total fat) (62). Although the wafers sold in Lebanese supermarkets and tested recently contained the lowest levels of TFAs (3.25/100 g of total fat) compared to the oldest values in 2015 (14.8/100 g of total fat) (36), it appears that a locally manufactured wafer contained 6/100 g of total fat of TFAs, which exceeded the WHO recommendations. Compared to Morocco (2.1/100 g of total fat) (63) and Jordan (4.8/100 g of total fat) (61), the TFAs average of traditional dishes was relatively low (0.92/100 g of total fat) (Appendix-Table I).

### Trans Fatty Acid Intake in Lebanon Compared to Eastern Mediterranean Countries

According to Appendix I, few studies reported that TFAs consumption levels in EMR and estimates were found to range between 0.28 % EI in Tunisia (based on the national survey conducted among adults in 2005) (18) and to reach as high as 6.5% EI in Egypt (64). The value of TFAs intake reported in Lebanon in 2016 (2.3% EI) (65) was relatively high compared to the WHO recommendations of 1% EI. On the other hand, the proportion of CHD deaths due to TFA intake was low in Lebanon (1.02%) compared to the mean of Eastern Mediterranean countries proportions (2.61%). The highest mortality by CHD due to TFA was observed in Egypt followed by Iran and Pakistan (16). Iran and Pakistan implemented mandatory limits to control TFA in foods along with Bahrain, Kuwait, Saudi Arabia, and Jordan in which the death by CHD due to TFA is among the lowest in the region (1.02%).

### Comparison Between Lebanese Market Basket Investigation and Other Global and Regional Market Investigations

According to many studies, there was an impact of TFAs and PHFAs labeling on reducing the burden of CVDs due to IP-TFAs (66). According to an unpublished study conducted by our team, 32% only of the products available in the Lebanese markets reported IP-TFAs on their labels (data not shown). Our finding came to hand in hand with Kamel et al. (67), in which 181 food products were sampled from local supermarkets in Saudi Arabia and showed that one-third of the products mentioned IP-TFAs on the nutrition label. Moreover, while the majority of the investigated samples in our project had low levels of IP-TFAs, up to 14 g of TFAs per 100 g of food was observed in certain oils and fats sold at the Lebanese markets. Our findings, concerning the range of IP-TFAs in market products, were relatively low compared to the market investigations published in Stender et al. (68, 69).

### Investigation of the Country of Origin of Imported Food Products in Lebanon

Lebanon imported its food products from France (107,957$), Germany (98,250$), Turkey (97,015$), the United Kingdom (75,571$), Italy (70, 571$), Argentina (69,989$), Saudi Arabia (64, 332$), and the United States (57,785$). In addition, the main importation sources of butter, oils, and fats are Denmark, the Netherlands, France, Belgium, Ukraine, New Zealand, the United Kingdom, and Argentina (All of us deal with IP-TFA consumption's clinical aftermath and all of us should be interested in stopping this threat) (20). According to the nutrition labels of tested butter and margarine, the countries of origin from which all the butter and margarine were imported to Lebanon were Turkey (*n* = 5), Egypt (*n* = 4), Malaysia (*n* = 3), Saudi Arabia (*n* = 1), Sri Lanka (*n* = 3), UAE (*n* = 1), Netherland (*n* = 2), Belgium (*n* = 3), France (*n* = 4), Italy (*n* = 1), Ukraine (*n* = 1), Germany (*n* = 2), and Denmark (*n* = 2) (data not shown). Among all these countries, 33 percent (5 countries over 15) are implementing mandatory national limits and adopting monitoring mechanisms for mandatory of IP-TFAs limits. On the other hand, in the remaining countries, the best-practice TFAs policy passed but was not yet in effect (16). Lebanon, long considered a middle-income country, is rapidly sinking into poverty as it faces a triple shock from the unprecedented economic crisis, the impact of COVID-19 on employment and public health, and the consequences of the Beirut port explosion. Despite that, the actual relative impact of IP-TFAs exposure on heart disease mortality in Lebanon is limited but unambiguously still considerable. The findings in our report highlight the importance of controlling the importation of food products from countries controlling IP-TFA levels in food to avoid sinking Lebanese markets with IP-TFA-rich food products (20), both of which are often ultra-processed, unhealthy, and rich in IP-TFAs. Therefore, this population group is at higher risk of TFA-attributable CVDs.

This study presents some limitations. First, there are many challenges facing the laboratories in Lebanon concerning the testing of IP-TFA, and the lack of standards limits testing other forms of isomers. Second, the food products compared between regions were compared in terms of food groups and not in terms of brands. Moreover, the comparison between traditional dishes or Arabic sweets omits the cooking preparations and ingredients. Third, in the current study, the WHO technique was followed to test the IP-TFA levels in foods tested; however, this was not always reported in many other countries.

## Conclusion

For the first time in Lebanon, a valid TFA database is available and ready to be used by healthcare providers. There is more than enough convincing evidence that a high IP-TFAs intake is detrimental to cardiovascular health. Fortunately, this problem in Lebanon is fairly easy to solve *via* proper legislation. However, the persistence of food products with high IP-TFA levels in Lebanon means that subgroups of the Lebanese population, mainly vulnerable and food-insecure people, are threatened by high levels of TFAs due to frequent consumption of risky products. The inauguration and implementation of policies to curtail IP-TFAs in Lebanon may therefore be legitimized, and such efforts should underline added fats and packaged foods due to the economic crises in which people opt to select cheap oils, including butter, ghee, and margarine instead of vegetable oils. We all deal with the clinical consequences of IP-TFA consumption, and we should all be engaged in putting an end to this threat. Although trans fatty acids have a small impact on heart disease mortality in Lebanon, they are unquestionably significant. The persistence of food products with high quantities of trans fatty acids poses a health risk to Lebanese citizens. Fortunately, proper laws in Lebanon can easily remedy this situation.

## Data Availability Statement

The original contributions presented in the study are included in the article/Supplementary Material, further inquiries can be directed to the corresponding author/s.

## Author Contributions

MH involved in conceptualization, data curation, formal analysis, investigation, methodology, project administration, supervision, validation, and writing the original draft preparation. EZ was involved in conceptualization, methodology, project administration, writing the original draft preparation, and writing the review and editing. AR and IS involved in methodology, project administration, supervision, and writing the review and editing. CI was involved in writing the review and editing. AA-J involved in data curation, methodology, project administration, supervision, validation, and writing the review and editing. All authors contributed to the article and approved the submitted version.

## Funding

This project was funded by RESOLVES to SAVE LIVES.

## Author Disclaimer

The authors alone are responsible for the views expressed in this article, and they do not necessarily represent the views, decisions, or policies of WHO or the other institutions with which the authors are affiliated.

## Conflict of Interest

The authors declare that the research was conducted in the absence of any commercial or financial relationships that could be construed as a potential conflict of interest.

## Publisher's Note

All claims expressed in this article are solely those of the authors and do not necessarily represent those of their affiliated organizations, or those of the publisher, the editors and the reviewers. Any product that may be evaluated in this article, or claim that may be made by its manufacturer, is not guaranteed or endorsed by the publisher.
