# Supplementary material for: Assessment of Industrially Produced Trans Fatty Acids in Traditional Dishes, Arabic Sweets, and Market Food Products and Its Risks on Non-communicable Diseases in Lebanon
Source: Front Nutr. 2021 Oct 21;8:727548. doi: 10.3389/fnut.2021.727548 (PMC8566673; doi:10.3389/fnut.2021.727548)
Supplement: Supplementary file 1 [file Table_1.docx]

|  | TFA Intake per country | | | TFA Content in Food groups per countries | | | | | | | | | | | |
| --- | --- | --- | --- | --- | --- | --- | --- | --- | --- | --- | --- | --- | --- | --- | --- |
|  |  |  |  | **(Average; range: g/100 g of total fat)** | | | | | | | | | | | |
|  | **%EI** | **g/day** | **Studies per year** | | **Arabic sweets** | **Bakery products** | **Biscuits** | **Butter and/or Margarines** | **Cake** | **Cereals based foods** | **Chocolate** | **Coffee and instant coffee** | **Creamer** | **Croissant** |  |
| Afghanistan | 1.3* | - | - | | - | - | - | - | - | - | - | - | - | - |  |
| Bahrain | 3.2* | - | - | | - | - | - | - | - | - | - | - | - | - |  |
| Djibouti | 0.8* | - | - | | - | - | - | - | - | - | - | - | - | - |  |
| Egypt | 6.5* | - | - | | - | - | - | - | - | - | - | - | - | - |  |
| Iran | 4.2  (2.48*)**(2007)** | 12.3  **(2007)** | **2013** | | - | - | - | 2.92  0.46-5.40 | - | - | - | - | - | - |  |
|  |  |  | **2014** | | - | - | - | 6.91  0.48-23.97 | - | - | - | - | - | - |  |
|  |  |  | **2015** | | - | - | 12.86 | - | 6.95 | - | 1.24 | - | 13.94 | - |  |
|  |  |  | **2016** | | - | - | - | 6.1  0.47-11.63 | - | - | - | - | - | - |  |
| Iraq | 1.8* | - | - | | - | - | - | - | - | - | - | - | - | - |  |
| Jordan | 0.7 (1.6*) | - | **2012** | | 4.08  3.4-4.6 | 2.46±0.97  1.35-4.4 | 2.82±2.09  0.73-7 | 4.34±2.92  0.04-6.2 | 3.4  3.05-4.1 | 2.51±0.78  1.95-3 | - | - | - | - |  |
| Kuwait | 1.86* | - | - | | - | - | - | - | - | - | - | - | - | - |  |
| Lebanon | 2.3 (1.6*)  **(2016)** | 6.1  **(2016)** | - | | - | - | - | - | - | - | - | - | - | - |  |
|  | - | - | **Current study** | | 0.5  <0.1-2.4 | 0.6  <0.1-2.7 | 0.17  <0.1-0.3 | 1.23  <0.1-11.8 | 1.3  <0.1-2.6 | 0.15  <0.1-0.3 | 0.03  <0.1-0.1 | 0.25  0.2-0.3 | - | 0.4  <0.1-0.7 |  |
|  | - | - | **2015** | | - | - | 9.7±8.1  0.7-22.3 | - | 5.8±3.2  1.8-11.7 | - | - | - | - | 7.8±1.9  5.6-9.1 |  |
| Libya | 1.6* | - | - | | - | - | - | - | - | - | - | - | - | - |  |
| Morocco | 1.1* | - | **2015** | | - | - | - | 9.1-21.7 | - | - | - | - | - | - |  |
| Oman | 1.9* | - | - | | - | - | - | - | - | - | - | - | - | - |  |
| Pakistan | 5.8* | - | **2020** | | - | - | 9.3-34.9 | 1.45-34.8 | 12.02 | 14.40-16.30 | 4.56-8.49 | - | - | - |  |
| Palestine | 1.9* | - | - | | - | - | - | - | - | - | - | - | - | - |  |
| Qatar | 1.7* | - | - | | - | - | - | - | - | - | - | - | - | - |  |
| Saudi Arabia | 1.1* | - | **2013** | | - | - | - | 3.9  0.2-8.3 | - | - | - | - | - | - |  |
| Somalia | 0.8* | - | - | | - | - | - | - | - | - | - | - | - | - |  |
| Sudan | 0.2(1.1*)  **(2006)** | - | - | | - | - | - | - | - | - | - | - | - | - |  |
| Syria | 1.5* | - | - | | - | - | - | - | - | - | - | - | - | - |  |
| Tunisia | 0.28(1.1*)  **(2009-2010** | - | **2019** | | - | - | - | 5.3  1.01-9.9 | - | - | - | - | - | - |  |
| UAE | 0.8 (1.10*)  **(2014)** | - | - | | - | - | - | - | - | - | - | - | - | - |  |
| Yemen | 1.5* | - | - | | - | - | - | - | - | - | - | - | - | - |  |

***Based on Bayesian model (Micha et al, 2014)**

|  | TFA Content in Food groups per countries | | | | | | | | |
| --- | --- | --- | --- | --- | --- | --- | --- | --- | --- |
|  | **(Average; range: g/100 g of total fat)** | | | | | | | | |
|  | **Studies per year** | **Edible oils** | **Fast foods** | **French fries** | **Milk and dairy products** | **Nuts and seeds** | **Pie** | **Pastries** | **Potato chips** |
| Afghanistan | - | - | - | - | - | - | - | - | - |
| Bahrain | - | - | - | - | - | - | - | - | - |
| Djibouti | - | - | - | - | - | - | - | - | - |
| Egypt | - | - | - | - | - | - | - | - | - |
| Iran | **2013** | 0.45  0.11-1.61 | - | - | - | - | - | - | - |
|  | **2014** | - | - | - | - | - | - | - | - |
|  | **2015** | - | - | - | - | - | - | - | 0.61 |
|  | **2016** | 0.72  0.07-2 | - | - | - | - | - | - | - |
| Iraq | - | - | - | - | - | - | - | - | - |
| Jordan | **2012** | 0.61±0.58  0.13-1.32 | 4.16±1.34  1.1-8.12 | 3.63±3.02  1.49-5.76 | 3.87±0.59  2.39-4.97 | - | - | - | 1.47±0.45  1.11-3.95 |
| Kuwait | - | - | - | - | - | - | - | - | - |
| Lebanon | - | - | - | - | - | - | - | - | - |
|  | **Current study** | <0.1  <0.1 | - | - | - | 0.4  <0.1-0.7 |  | 1.85  1.7-2 | 0.2  <0.1-0.3 |
|  | **2015** | - | - | - | - | - | - | - | - |
| Libya | - | - | - | - | - | - | - | - | - |
| Morocco | **2015** |  | 1.6±1.1  0.75-2.66 | - | - | - | - | - | - |
| Oman | - | - | - | - | - | - | - | - | - |
| Pakistan | **2020** | 0.96  0.45-1.63 |  | 10-24.0 |  |  |  | 3.92-10.17 | 0.4-26 |
| Palestine | - | - | - | - | - | - | - | - | - |
| Qatar | - | - | - | - | - | - | - | - | - |
| Saudi Arabia | **2013** | - | - | - | - | - | - | - | - |
| Somalia | - | - | - | - | - | - | - | - | - |
| Sudan | - | - | - | - | - | - | - | - | - |
| Syria | - | - | - | - | - | - | - | - | - |
| Tunisia | **2019** | 0.73  0.18-1.29 | 0.9±0.26 | - | - | - | 12.27±0.88 | - | - |
| UAE | - | - | - | - | - | - | - | - | - |
| Yemen | - | - | - | - | - | - | - | - | - |

|  | TFA Content in Food groups per countries | | | | | | | | |
| --- | --- | --- | --- | --- | --- | --- | --- | --- | --- |
|  | **(Average; range: g/100 g of total fat)** | | | | | | | | |
|  | **Studies per countries** | **Popcorn** | **Sausages and Luncheon meats** | **Sesame products** | **Snacks** | **Tuna**  **packs** | **Traditional**  **dishes** | **Wafers** |  |
| Afghanistan | - | - | - | - | - | - | - | - |  |
| Bahrain | - | - | - | - | - | - | - | - |  |
| Djibouti | - | - | - | - | - | - | - | - |  |
| Egypt | - | - | - | - | - | - | - | - |  |
| Iran | **2013** | - | - | - | - | - | - | - |  |
|  | **2014** | - | - | - | - | - | - | - |  |
|  | **2015** | - | - | - | 0.52 | - | - | - |  |
|  | **2016** | - | - | - | - | - | - | - |  |
| Iraq |  | - | - | - | - | - | - | - |  |
| Jordan | **2012** | 20.38±20.18  1.26-41.1 | 3.84±1.04  2.57-5.5 | - | 3.93±8.85  0.73-41.11 | - | 4.8  1.5-8.1 | - |  |
| Kuwait | - | - | - | - | - | - | - | - |  |
| Lebanon | - | - | - | - | - | - | - | - |  |
|  | **Current study** | - | - | 0.6  0.1-1.3 | - | 0.45  0.3-0.6 | 0.92  <0.1-1.9 | 3.25  <0.1-6.5 |  |
|  | **2015** | - | - | - | - | - | - | 14.8±10.1  4.3-25.8 |  |
| Libya | - | - | - | - | - | - | - | - |  |
| Morocco | **2015** | - | - | - | - | - | 2.1±1.9  0.29-6.3 | - |  |
| Oman |  | - | - | - | - | - | - | - |  |
| Pakistan | **2020** | - | - | - | - | - | - | - |  |
| Palestine |  | - | - | - | - | - | - | - |  |
| Qatar |  | - | - | - | - | - | - | - |  |
| Saudi Arabia | **2013** | - | - | - | - | - | - | - |  |
| Somalia |  | - | - | - | - | - | - | - |  |
| Sudan |  | - | - | - | - | - | - | - |  |
| Syria |  | - | - | - | - | - | - | - |  |
| Tunisia | **2019** | - | - | - | - | - | - | - |  |
| UAE |  | - | - | - | - | - | - | - |  |
| Yemen |  | - | - | - | - | - | - | - |  |

**Table S1- Trans fatty acid intake and trans fatty acid content in 100 g of total fat per food groups among Eastern Mediterranean countries**
